# Recent Rapid Rise of a Permethrin Knock Down Resistance Allele in *Aedes aegypti* in México

**DOI:** 10.1371/journal.pntd.0000531

**Published:** 2009-10-13

**Authors:** Gustavo Ponce García, Adriana E. Flores, Ildefonso Fernández-Salas, Karla Saavedra-Rodríguez, Guadalupe Reyes-Solis, Saul Lozano-Fuentes, J. Guillermo Bond, Mauricio Casas-Martínez, Janine M. Ramsey, Julián García-Rejón, Marco Domínguez-Galera, Hilary Ranson, Janet Hemingway, Lars Eisen, William C. Black

**Affiliations:** 1 Laboratorio de Entomología Médica, Facultad de Ciencias Biológicas, Universidad Autónoma de Nuevo León, San Nicolás de los Garza, Nuevo León, México; 2 Department of Microbiology, Immunology and Pathology, Colorado State University, Fort Collins, Colorado, United States of America; 3 Centro Regional de Investigación en Salud Pública, Instituto Nacional de Salud Pública, Tapachula, Chiapas, México; 4 Universidad Autónoma de Yucatán, Laboratorio de Arbovirología, Centro de Investigaciones Regionales Dr. Hideyo Noguchi, Universidad Autónoma de Yucatán, Mérida, Yucatán, México; 5 Servicios Estatales de Salud de Quintana Roo, Servicios Estatales de Salud de Quintana Roo, Chetumal, Quintana Roo, México; 6 Vector Group, Liverpool School of Tropical Medicine, Liverpool, United Kingdom; Mahidol University, Thailand

## Abstract

**Background:**

*Aedes aegypti*, the ‘yellow fever mosquito’, is the primary vector to humans of dengue and yellow fever flaviviruses (DENV, YFV), and is a known vector of the chikungunya alphavirus (CV). Because vaccines are not yet available for DENV or CV or are inadequately distributed in developing countries (YFV), management of *Ae. aegypti* remains the primary option to prevent and control outbreaks of the diseases caused by these arboviruses. Permethrin is one of the most widely used active ingredients in insecticides for suppression of adult *Ae. aegypti*. In 2007, we documented a replacement mutation in codon 1,016 of the voltage-gated sodium channel gene (*para*) of *Ae. aegypti* that encodes an isoleucine rather than a valine and confers resistance to permethrin. Ile1,016 segregates as a recessive allele conferring knockdown resistance to homozygous mosquitoes at 5–10 µg of permethrin in bottle bioassays.

**Methods and Findings:**

A total of 81 field collections containing 3,951 *Ae. aegypti* were made throughout México from 1996 to 2009. These mosquitoes were analyzed for the frequency of the Ile1,016 mutation using a melting-curve PCR assay. Dramatic increases in frequencies of Ile1,016 were recorded from the late 1990's to 2006–2009 in several states including Nuevo León in the north, Veracruz on the central Atlantic coast, and Yucatán, Quintana Roo and Chiapas in the south. From 1996 to 2000, the overall frequency of Ile1,016 was 0.04% (95% confidence interval (CI95) = 0.12%; n = 1,359 mosquitoes examined). The earliest detection of Ile1,016 was in Nuevo Laredo on the U.S. border in 1997. By 2003–2004 the overall frequency of Ile1,016 had increased ∼100-fold to 2.7% (±0.80% CI95; n = 808). When checked again in 2006, the frequency had increased slightly to 3.9% (±1.15% CI95; n = 473). This was followed in 2007–2009 by a sudden jump in Ile1,016 frequency to 33.2% (±1.99% CI95; n = 1,074 mosquitoes). There was spatial heterogeneity in Ile1,016 frequencies among 2007–2008 collections, which ranged from 45.7% (±2.00% CI95) in the state of Veracruz to 51.2% (±4.36% CI95) in the Yucatán peninsula and 14.5% (±2.23% CI95) in and around Tapachula in the state of Chiapas. Spatial heterogeneity was also evident at smaller geographic scales. For example within the city of Chetumal, Quintana Roo, Ile1,016 frequencies varied from 38.3%–88.3%. A linear regression analysis based on seven collections from 2007 revealed that the frequency of Ile1,016 homozygotes accurately predicted knockdown rate for mosquitoes exposed to permethrin in a bioassay (R^2^ = 0.98).

**Conclusions:**

We have recorded a dramatic increase in the frequency of the Ile1,016 mutation in the voltage-gated sodium channel gene of *Ae. aegypti* in México from 1996 to 2009. This may be related to heavy use of permethrin-based insecticides in mosquito control programs. Spatial heterogeneity in Ile1,016 frequencies in 2007 and 2008 collections may reflect differences in selection pressure or in the initial frequency of Ile1,016. The rapid recent increase in Ile1,016 is predicted by a simple model of positive directional selection on a recessive allele. Unfortunately this model also predicts rapid fixation of Ile1,016 unless there is negative fitness associated with Ile1,016 in the absence of permethrin. If so, then spatial refugia of susceptible *Ae. aegypti* or rotational schedules of different classes of adulticides could be established to slow or prevent fixation of Ile1,016.

## Author Summary

Pyrethroid insecticides prolong the opening of voltage-dependent sodium channels in insect nerves to produce instant paralysis and “knock-down.” Many insects have evolved knock-down resistance through nonsynonymous mutations that reduce pyrethroid binding in the channels. In 2006 we discovered one such mutation in the arbovirus mosquito vector *Aedes aegypti*, called Ile1,016, that confers very high knockdown resistance to the pyrethroid insecticide permethrin in mosquitoes homozygous for this mutation. We examined collections of *Ae. aegypti* from México during 1996–2009 and found that the overall Ile1,016 frequency increased from <0.1% in 1996–2000, to 2%–5% in 2003–2006, to 38.3%–88.3% in 2007–2009 depending upon collection location. We also demonstrate a strong linear relationship between the frequency of Ile1,016 homozygotes and knockdown rate in bioassays and speculate that widespread use of permethrin-based insecticides in México may be impacting the frequency of Ile1,016. Such a rapid increase is predicted by a simple model of positive directional selection acting on a recessive allele. Unfortunately this model also predicts rapid fixation of Ile1,016 unless there is a negative fitness associated with Ile1,016 in the absence of permethrin and if insecticidal pressure can be reduced.

## Introduction

*Aedes aegypti*, the ‘yellow fever mosquito’, is the primary vector to humans of dengue and yellow fever flaviviruses (DENV, YFV) [1]–[3]. Vaccines are not yet available against DENV [4] and, despite the presence of a safe and effective YFV vaccine [5]–[7], the World Health Organization estimates there are 200,000 cases and 30,000 deaths attributable to yellow fever each year [8]. The principal means to reduce transmission of these arboviruses has therefore been through control or eradication of *Ae. aegypti* [9]. Historic eradication campaigns that combined source reduction to remove larval development sites with use of dichloro-diphenyl-trichloroethane (DDT) to kill adults were successful in eliminating the mosquito and its associated arboviruses, especially in the Americas, but these programs were not sustained and *Ae. aegypti* and DENV re-emerged in force [10],[11]. In recent decades, pyrethroid insecticides have played a major global role in the control of *Ae. aegypti* adults, often in combination with the organophosphate insecticide temephos to control immatures. However, the evolution of resistance to these and other insecticides in *Ae. aegypti* may compromise the effectiveness of control programs [12]–[14].

Since 1950, operational vector control programs in México have used a series of insecticides to control mosquito vectors and reduce arbovirus and malaria transmission (Official Regulations of México, NOM-032-SSA) [12]. The organochlorine insecticide DDT was used extensively for indoor house spraying from 1950–1960 and was still used in some locations until 1998. Organophosphate insecticides with malathion as the active ingredient were later used for ultra-low volume (ULV) space spraying of wide areas from 1981 to 1999. In 2000, vector control programs in México then switched to permethrin-based insecticides for adult control. This has provided prolonged and intense selection pressure for resistance evolution in *Ae. aegypti*. Indeed, pyrethroid insecticides with active ingredients such as permethrin, deltamethrin, resmethrin and sumithrin are now commonly applied across the world to kill adult mosquitoes and reduce the burden of mosquito-borne diseases. The future global use of bednets, curtains and other household items treated with pyrethroids for personal protection will likely increase dramatically [15]–[18]. This underscores the critical need to monitor and manage resistance to pyrethroid insecticides to maintain their use for vector control.

Pyrethroids act by structure-related interactions with specific regions of voltage-dependent sodium channels that prolong the opening of these channels, and produce instant paralysis [19]. Nervous system stimulation proceeds from excitation to convulsions and tetanic paralysis. Metabolic resistance and target site insensitivity are both major forms of pyrethroid resistance [19],[20]. ‘Knockdown resistance’ (kdr) is a generic term applied to insects that fail to lose coordinated activity immediately following pyrethroid exposure. Typically kdr is unaffected by synergists that inhibit esterases and monooxygenases. Instead kdr arises through nonsynonymous mutations in the voltage-gated sodium channel transmembrane gene (orthologue of the *para*lysis locus in *Drosophila melanogaster*) [21] that reduce pyrethroid binding. Kdr usually limits the effectiveness of pyrethroids to varying degrees depending on whether the insecticide contains a descyano-3-phenoxybenzyl alcohol (type I pyrethroid) or an α-cyano-3-phenoxybenzyl alcohol (type II). Thus detection of kdr in the field may have severe consequences for sustained use of pyrethroids in mosquito control.

A homology model of the housefly *para* protein was developed [22] to predict the location of binding sites for the pyrethroid, fenvalerate and for DDT. The model addressed the state-dependent affinity of pyrethroid insecticides, their mechanism of action and the role of mutations in the channel that are known to confer insecticide resistance. Specifically, the sodium channel was modeled in an open conformation with the insecticide binding site located in the hydrophobic cavity delimited by the domain II subunit 4 (IIS4) - IIS5 linker and the IIS5 and IIS6 helices. Five novel mutations in IIS6, one in IIS5 and one in the P loop were described in the *para* orthologue in *Ae. aegypti* [23]. Assays on larvae from strains bearing these mutations indicated reduced nerve sensitivity to permethrin inhibition. Two of these mutations occurred in codons Ile1,011 and Val1,016 in exons 20 and 21, respectively. A transition in the third position of Ile1,011 encoded a Met1,011 replacement and a transversion in the second position of Val1,016 encoded a Gly1,016 replacement. This same region of IIS6 was later screened in 1,318 mosquitoes in 32 additional strains; 30 from throughout Latin America [24]. The Gly1,016 allele was never detected in Latin America and instead we found two new mutations in these same codons. A transition in the first position of codon 1,011 encodes a valine replacement while a transition in the first position of codon 1,016 encoded an isoleucine replacement. We developed melting curve PCR assays for these four mutations. Selection experiments, one with deltamethrin on a field strain from Santiago de Cuba and another with permethrin on a strain from Isla Mujeres, México rapidly increased the frequency of the Ile1,016 allele [25]. In bioassays of F_3_ offspring arising from crosses of permethrin susceptible Val1,016 homozygous parents and permethrin resistant Ile1,016 homozygous parents, Ile1,016 segregated as a recessive allele conferring knockdown resistance to homozygous mosquitoes at 5–10 µg permethrin in bottle bioassays, 4.3–14.0% resistance in heterozygous mosquitoes [24]. All Val1,016 homozygous mosquitoes died.

Herein we report on an analysis of the frequency of the Ile1,016 mutation in 3,808 *Ae. aegypti* from 78 collections made from 1996–2008 throughout México. The overall frequency was 0.04% from 1996–2001, had climbed to 2.7% by 2003–2004, and increased only slightly to 3.6% by 2006. Then, as would be expected with a recessive allele, Ile1,016 frequency rapidly increased to 33% in 2007–2009. We also document a great deal of spatial heterogeneity in Ile1,016 frequency during 2007–2009. A linear regression analysis based on seven collections from 2007 revealed that the frequency of Ile1,016 homozygotes accurately predicted knockdown rate for mosquitoes exposed to permethrin in a bioassay (R^2^ = 0.98). These results have led us to speculate that widespread use of permethrin-based insecticides in México from 2000–2008 may have resulted in rapidly increasing frequencies of the Ile1,016 mutation in *Ae. aegypti*. Potential implications and solutions for operational vector control are discussed.

## Materials and Methods

### *Aedes aegypti* collections and extraction of DNA

**Figure 1:**
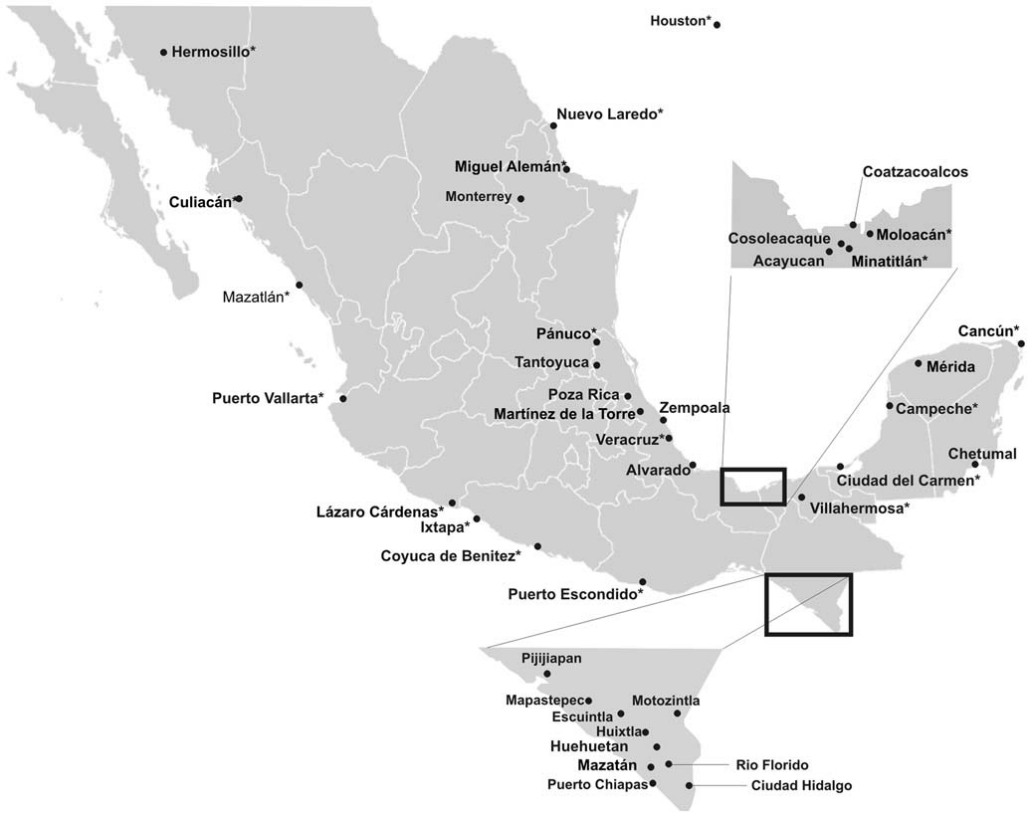
Locations of collection sites in México at which Ile1,016 frequencies for *Aedes aegypti* were estimated. Only single collections were made in those cities followed by *.

Table 1 lists the cities and years of collection for *Aedes aegypti*, and city locations are mapped in Figure 1. Single collections were made in those cities marked by * in Figure 1. Superscripts next to the year of the collection in Table 1 indicate in which of three prior studies [26]–[28] samples were collected. Collections from 2006–2009 have not been included in any prior studies. At each collection site, we collected immatures from at least 30 different containers in each of three different areas located at least 100 m apart. This included water storage containers and discarded trash containers such as plastic pails, tires, and cans. Larvae were returned to the laboratory where they were reared to adults and then identified to species [29]. All mosquitoes were stored at −80°C prior to examination for presence of Ile1,016. DNA was obtained from individual adults by salt extraction [30], suspended in 300 µl of TE buffer (10 mM Tris-HCl, 1 mM EDTA pH 8.0), and stored at −80°C.

**Table 1:** Locations, collection years, sample sizes and numbers of mosquitoes of each genotype.

State	Year	Sample Size	AA	AG	GG	Frequency Ile1,016	Wald 95% CI	F_IS_
City								
**Texas (U.S.A.)**								
Houston	1998^a^	47	0	0	47	0.000	(0.00–0.03)	-
**Tamaulipas**								
Nuevo Laredo	1997^a^	50	0	1	49	0.010	(0.00–0.06)	−0.010
Miguel Alemán	1998^a^	47	0	0	47	0.000	(0.00–0.03)	-
**Nuevo León**								
Monterrey	1996^a^	47	0	0	47	0.000	(0.00–0.03)	-
Monterrey	2008	44	8	28	8	0.500	(0.40–0.60)	−0.273
**Veracruz**								
Pánuco	2003^c^	50	1	18	31	0.200	(0.13–0.29)	−0.125
Tantoyuca	2003^c^	50	0	0	50	0.000	(0.00–0.06)	-
	2004^c^	33	2	0	31	0.060	(0.02–0.15)	1.000***
	2008	50	6	26	18	0.380	(0.29–0.48)	−0.104
Poza Rica	2003^c^	46	0	0	46	0.000	(0.00–0.03)	-
	2004^c^	50	1	1	48	0.030	(0.01–0.09)	0.656***
	2008	50	26	18	6	0.700	(0.60–0.78)	0.143
Martínez de la Torre	2003^c^	50	0	0	50	0.000	(0.00–0.06)	-
	2008	50	6	31	13	0.430	(0.34–0.53)	−0.265
Zempoala	2003^c^	47	0	0	47	0.000	(0.00–0.03)	-
	2004^c^	47	0	0	47	0.000	(0.00–0.03)	-
Veracruz	2008	50	19	23	8	0.610	(0.51–0.70)	0.033
Alvarado	2003^c^	50	0	7	43	0.070	(0.03–0.14)	−0.075
	2004^c^	47	0	0	47	0.000	(0.00–0.03)	-
Acayucan	2003^c^	47	0	0	47	0.000	(0.00–0.03)	-
	2004^c^	47	0	0	47	0.000	(0.00–0.03)	-
Cosoleacaque	2003^c^	50	0	2	48	0.020	(0.01–0.07)	−0.020
	2008	50	6	23	21	0.350	(0.26–0.45)	−0.011
Minatitlán	2003^c^	50	0	5	45	0.050	(0.02–0.11)	−0.053
	2004^c^	47	0	0	47	0.000	(0.00–0.03)	-
Coatzacoalcos	2003^c^	47	0	0	47	0.000	(0.00–0.03)	-
	2004^c^	50	0	2	48	0.020	(0.01–0.07)	−0.020
	2008	50	0	27	23	0.270	(0.19–0.36)	−0.370**
Moloacán	1999^a^	47	0	0	47	0.000	(0.00–0.03)	-
**Tabasco**								
Villahermosa	1998^a^	47	0	0	47	0.000	(0.00–0.03)	-
	1999^a^	47	0	0	47	0.000	(0.00–0.03)	-
**Campeche**								
Ciudad del Carmen	1998^a^	47	0	0	47	0.000	(0.00–0.03)	-
Campeche	1998^a^	47	0	0	47	0.000	(0.00–0.03)	-
**Yucatán**								
Mérida	1999^a^	47	0	0	47	0.000	(0.00–0.03)	-
Mérida - Center	1999^a^	47	0	0	47	0.000	(0.00–0.03)	-
Mérida - East	1999^a^	47	0	0	47	0.000	(0.00–0.03)	-
Mérida - North	1999^a^	47	0	0	47	0.000	(0.00–0.03)	-
Mérida - South	1999^a^	37	0	0	37	0.000	(0.00–0.04)	-
Mérida - West	1999^a^	47	0	0	47	0.000	(0.00–0.03)	-
Mérida - North	2007	50	14	26	10	0.540	(0.44–0.63)	−0.047
Mérida - South	2007	50	12	29	9	0.530	(0.43–0.62)	−0.164
Mérida - Dzununcan	2009	47	5	13	29	0.240	(0.17–0.34)	0.252
Mérida - San Jose' Tzal	2009	48	9	21	18	0.410	(0.31–0.51)	0.093
Mérida – Cholul	2009	48	1	35	12	0.390	(0.29–0.49)	−0.539***
**Quintana Roo**								
Cancún	1999^a^	94	0	0	94	0.000	(0.00–0.03)	-
Chetumal-Center	1999^a^	94	0	0	94	0.000	(0.00–0.03)	-
Chetumal-North	1999^a^	94	0	0	94	0.000	(0.00–0.03)	-
Chetumal-Calderitas	2007	30	6	11	13	0.380	(0.27–0.51)	0.224
Chetumal-Lagunitas	2007	30	24	5	1	0.880	(0.78–0.95)	0.191
Chetumal-Lázaro Cárdenas	2007	30	10	16	4	0.600	(0.47–0.71)	−0.111
Chetumal-Antorchistas	2008	30	3	15	12	0.350	(0.24–0.48)	−0.099
Chetumal-Solidaridad	2008	30	2	12	16	0.270	(0.17–0.39)	−0.023
**Chiapas**								
Ciudad Hidalgo	2006	45	0	16	29	0.180	(0.11–0.27)	−0.216
	2008	48	2	11	35	0.160	(0.10–0.24)	0.131
Motozintla	2006	48	0	0	48	0.000	(0.00–0.03)	-
	2008	47	0	2	45	0.020	(0.00–0.08)	−0.022
Rio Florido	1998^a^	94	0	0	94	0.000	(0.00–0.03)	-
	2006	47	0	4	43	0.040	(0.01–0.11)	−0.044
	2008	50	1	12	37	0.140	(0.08–0.22)	0.003
Puerto Chiapas	2006	48	0	0	47	0.000	(0.00–0.03)	-
	2008	40	0	8	32	0.100	(0.05–0.19)	−0.111
Mazatán	2006	48	0	9	39	0.090	(0.05–0.17)	−0.103
	2008	50	2	11	37	0.150	(0.09–0.23)	0.137
Huehuetán	2006	48	0	1	47	0.010	(0.00–0.06)	−0.011
	2008	50	1	8	41	0.100	(0.05–0.18)	0.111
Huixtla	2006	47	0	0	47	0.000	(0.00–0.03)	-
	2008	50	3	30	17	0.360	(0.27–0.46)	−0.302*
Escuintla	2006	46	0	7	39	0.080	(0.03–0.15)	−0.082
	2008	45	7	10	28	0.270	(0.19–0.37)	0.432**
Mapastepec	2006	48	0	0	48	0.000	(0.00–0.03)	-
	2008	50	0	5	45	0.050	(0.02–0.11)	−0.053
Pijijiapan	2006	48	0	0	48	0.000	(0.00–0.03)	-
	2008	50	0	10	40	0.100	(0.05–0.18)	−0.111
**Oaxaca**								
Puerto Escondido	1999^a^	47	0	0	47	0	(0.00–0.03)	-
**Guerrero**								
Coyuca de Benitez	1999^a^	47	0	0	47	0	(0.00–0.03)	-
Ixtapa	1999^a^	47	0	0	47	0	(0.00–0.03)	-
**Michoacán**								
Lázaro Cárdenas	1999^a^	47	0	0	47	0	(0.00–0.03)	-
**Jalisco**								
Puerto Vallarta	1999^a^	50	0	0	50	0	(0.00–0.06)	-
**Sinaloa**								
Mazatlán	1999^a^	47	0	0	47	0	(0.00–0.03)	-
Culiacán	1998^a^	47	0	0	47	0	(0.00–0.03)	-
**Sonora**								
Hermosillo	2000^b^	47	0	0	47	0	(0.00–0.03)	-
**81 collections**		3,951						

AA = Ile1,016 homozygotes, AG = Ile1,016/Val1,016 heterozygotes, GG = Val1,016 homozygotes for *Aedes aegypti* in México from 1996 to 2008. Also listed are the frequency of Ile1,016 at each site, the 95% confidence interval around that frequency and the F_IS_ estimate and its significance (*P<0.05, **P< 0.01, ***P<0.001).

a [27].

b [26].

c [74].

### Genotype determinations

Genotypes at the Ilel,016 locus were detected using allele specific PCR. Genotypes were determined in a single-tube reaction using two different “allele-specific” primers, each of which contained a 3′ nucleotide corresponding to one of the two alleles and a reverse primer that amplified both alleles. Allele specific primers were manufactured (Operon Inc., Huntsville, AL) with 5′ tails [31],[32] (shown in brackets below) that were designed to allow discrimination between SNP alleles based on size or melting temperature. The Valine allele specific primer was Val1,016 (5′-[GCGGGCAGGGCGGCGGGGGCGGGGCC]ACAAATTGTTTCCCACCCGCA CCGG-3′) and the isoleucine allele specific primer was Ile1,016 (5′-[GCGGGC]ACAAATTGTT TCCCACCCGCACTGA-3′). Brackets indicate the portion of the primer added for melting curve PCR. The reverse primer was Ile1,016r 5′-GGATGAACCSAAATTGGACAAAAGC-3′ [24]. An intentional transversion mismatch was introduced three bases in from the 3′ end of allele specific primers to improve specificity and each allele specific primer differed by a transition at this site [33]. Melting curve PCR was performed as previously described [34].

### Statistical analysis of haplotype and allele frequencies

Ile1,016 frequencies (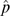) were calculated in each collection as twice the number of Ile1,016 homozygotes plus the number of Ile1,016 heterozygotes and then divided by twice the number of mosquitoes analyzed. Wright's inbreeding coefficient F_IS_ [35] was estimated as
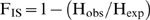
(1)Where H_obs_ is the observed number of heterozygotes and 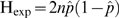 is the expected number of heterozygotes where *n* is the sample size and assuming Hardy-Weinberg proportions. The null hypothesis F_IS_ = 0 was tested using the formula [36]:
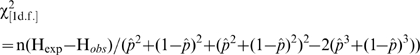
(2)The 95% confidence interval (CI95) around 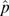 was calculated as the Wald interval:
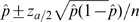
(3)which was then adjusted by adding half of the squared Z-critical value (1.96) to the numerator and the entire squared critical value to the denominator before computing the interval [37]. Fisher's model of natural selection [38] was used to estimate the expected trajectories for the Ile1,016 allele for a single population in which:
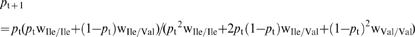
(4)where: *p* = Ile1,016 frequency in generation t. Following permethrin exposure w_Ile/Ile_ is the relative survival of Ile1,016 homozygotes, w_Ile/Val_ is the relative survival of Ile1,016 heterozygotes and w_Val/Val_ is the relative survival of Val1,016 homozygotes.

### Insecticidal bioassay

Knockdown rates were determined by releasing 40 adults, 3–4 days of age, into 250 mL Wheaton bottles in which the inside walls were coated with either 5.0 or 10.0 µg of permethrin (technical grade; Chem Services, West Chester, PA) [39]. Following a 1-hr exposure period, the number of inactive mosquitoes were recorded.

## Results

### Spatial and temporal trends in Ile1,016 frequency

Table 1 lists the location, collection years, sample sizes and numbers of mosquitoes of each genotype, the frequency of Ile1,016 at each site, the 95% confidence interval around that frequency and the F_IS_ estimate and its significance. If F_IS_ was significantly >0 then an excess of homozygotes was present while if F_IS_ was significantly <0 then an excess of heterozygotes was present. F_IS_ was significantly greater or less than zero in 6 of the 40 collections in which Ile1,016 was present. F_IS_ values>0 were recorded in three cases because of unexpected Ile1,016 homozygotes (Tantoyuca and Poza Rica 2004) or a general deficiency of heterozygotes (Escuintla 2008). In three cases, F_IS_ values<0 occurred because of an excess of heterozygotes (Huixtla and Coatzacoalcos 2008; Mérida – Cholul 2009).

**Figure 2:**
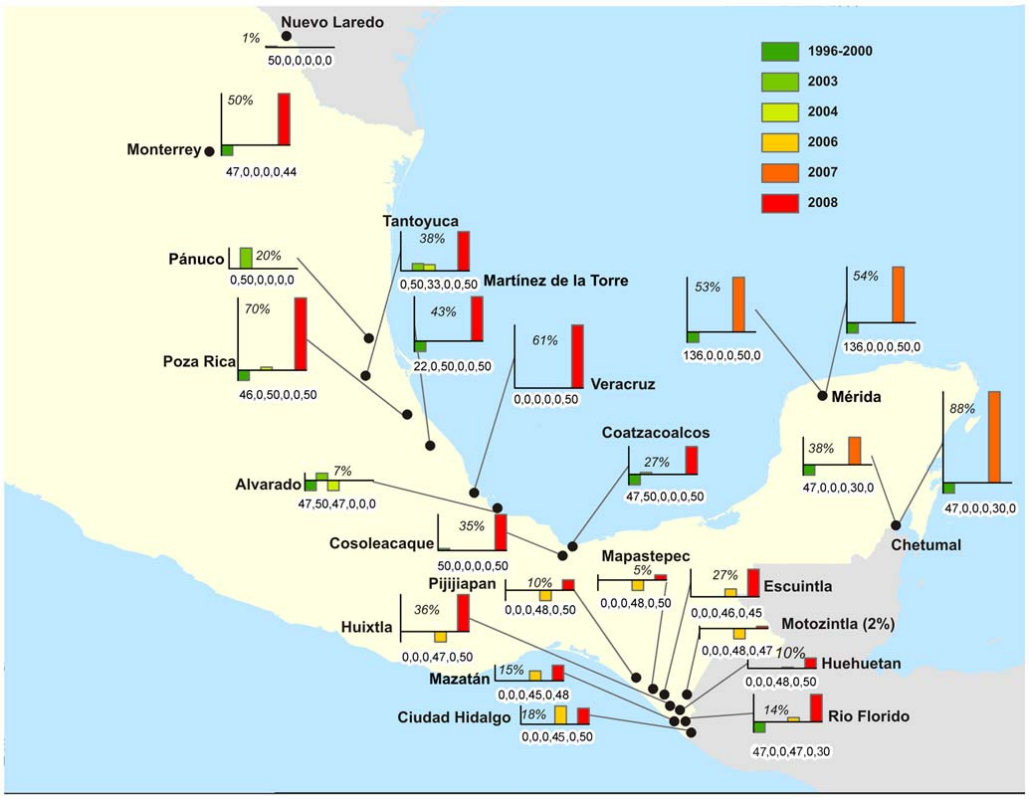
Frequencies of Ile1,016 for *Aedes aegypti* by time period for sites in México where the allele appeared at least once. Bars below the abscissa indicate that Ile1,016 wasn't detected. The numbers in each graph indicate the maximum frequency of Ile1,016 detected.

The map-based representation in Figure 2 shows frequencies of Ile1,016 by year of collection for all sites where the allele appeared at least once. Ile1,016 first appeared amongst our collections in Nuevo Laredo on the U.S. border in 1997 (Table 1, Figure 2). Overall frequency of Ile1,016 was very low, 0.04%, from 1996–2000 (CI95 = 0.12%; n = 1,359 mosquitoes examined). This included mosquitoes collected throughout México. No mosquitoes were collected in 2001–2002. In 2003–2004, collections were made exclusively in the state of Veracruz which is located along the central Atlantic coast of México. In 2003, Ile1,016 appeared in four collections with an overall frequency of 3.49% (1.18% CI95; n = 487 mosquitoes). Notably, the Ile1,016 frequency in one site, Pánuco, reached 20.0% in 2003 (0.12% CI95). In 2004, Ile1,016 appeared in only two collections with an overall frequency of 1.40% (0.99% CI95; n = 321 mosquitoes). No mosquitoes were collected in 2005.

In 2006, ten collections were made in the state of Chiapas in the far southwest. Ile1,016 appeared in five collections with a cumulative frequency of 3.91% (1.15% CI95; n = 473). In 2007, five collections were made in the states of Yucatán and Quintana Roo in the Yucatán Peninsula of southeastern México and Ile1,016 appeared in all collections with an overall frequency of 57.6% (4.94% CI95; n = 190). These collections also revealed that frequencies were not uniform within cities. For example, the frequency of Ile1,016 for collections within the city of Chetumal in Quintana Roo State ranged from 38.3% (11.97% CI95) in the Calderitas neighborhood to 88.3% (8.50% CI95) in the Lagunitas neighborhood.

In 2008, collections were made at six sites in Veracruz, the same ten sites in Chiapas as in 2006, two more sites in the city of Chetumal, and one site in Monterrey in Nuevo León State in northern México. In Veracruz, the overall frequency of Ile1,016 was 45.7% (3.97% CI95; n = 300) and, as for Chetumal in 2007, there was a great deal of spatial heterogeneity with site-specific Ile1,016 frequencies varying between 27.0% (8.62% CI95) in the city of Coatzacoalcos to 70.0% (8.87% CI95) in the city of Poza Rica. In Chiapas, the overall frequency of Ile1,016 was 14.5% (2.23% CI95; n = 480 mosquitoes) with site-specific frequencies varying between 5.0% (4.80% CI95) in the mountain town of Mapastepec to 36.0% (9.26% CI95) in the coastal city of Huixtla. In Chetumal, Ile1,016 varied between 35.0% (11.77% CI95) in the Antorchistas neighborhood to 26.7% (11.02% CI95) in the Solidaridad neighborhood. The frequency of Ile1,016 in Monterrey, was 50.0% (10.23% CI95). Only 3 collections were made in satellite villages surrounding Mérida in 2009. No comparisons were made of these collections because they are confounded by year and by their distance from other Mérida collections.

**Figure 3:**
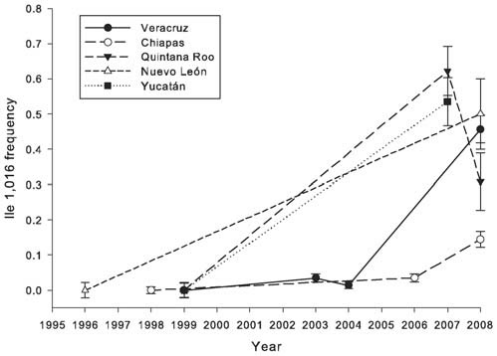
Overall frequency of Ile1,016 for *Aedes aegypti* by year in the states of Veracruz, Chiapas, Quintana Roo, Nuevo León, and Yucatán.

Figure 2 illustrates the general trend in México that the frequency of Ile1,016 has increased over the last 12 years in the states of Nuevo León, Veracruz, Yucatán, Quintana Roo and Chiapas. This is even more evident in Figure 3 in which the overall frequency of Ile1,016 in each state is plotted by year. It should be noted that although Figure 3 suggests that the Ile1,016 frequency in Chetumal, Quintana Roo, declined between 2007 and 2008, this could be related to sampling of sites in different neighborhoods in these years.

### Ile1,016 frequency and knockdown rates

**Figure 4:**
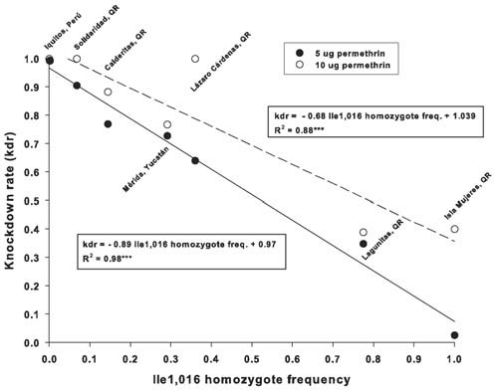
Linear regression analyses of the knockdown rate for *Aedes aegypti* in a bioassay as a function of the frequency of Ile1,016 homozygotes. This included seven mosquito collections and two concentrations of permethrin (5 and 10 µg per bottle).

Linear regression analysis was used to determine how well the frequency of Ile1,016 homozygotes in a collection of *Ae. aegypti* predicts knockdown rate in bioassays. Analyses included F_3_ adults from five collections from Chetumal, one collection from Isla Mujeres (northeast of Cancún) in which Ile1,016 is fixed, and one collection from Iquitos in Perú where Ile1,016 is absent. The Isla Mujeres strain arose from five generations of permethrin selection. We found strong associations between the frequency of Ile1,016 homozygotes and the knockdown rate for exposures of both 5 and 10 µg permethrin per bottle in the bioassay (R^2^ = 0.98 and 0.88, respectively; Figure 4). Exclusion of Isla Mujeres and Iquitos collections reduced the R^2^ values from 0.98 to 0.97 for the 5 µg concentration and from 0.88 to 0.76 for the 10 µg concentration.

### Expected trajectories for the Ile1,016 allele

**Figure 5:**
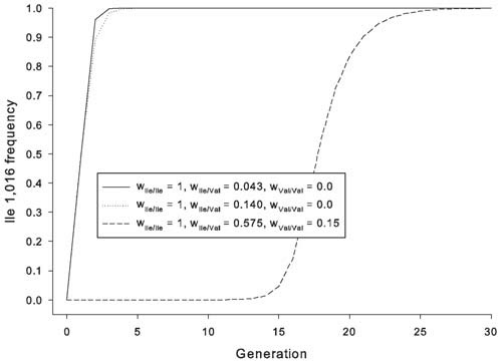
Expected trajectories for the Ile1,016 allele for a single population with an initial Ile1,016 frequency of 0.04% using Fisher's model of natural selection. Three scenarios are presented. The first two trajectories indicate a rapid fixation of Ile1,016 when Val1,016 is partially dominant (4–14% survival in heterozygous mosquitoes). The third scenario assumes that mosquitoes recover following knockdown. Based upon observation in the laboratory, all Ile1,016 homozygotes recover but 58% of heterozygotes and 15% of Val 1,016 homozygotes recover.

Figure 5 shows the expected trajectories for the Ile1,016 allele for a population with an initial Ile1,016 frequency of 0.04% (as observed in México during 1996–2000). The first two trajectories indicate a rapid fixation of Ile1,016 when Val1,016 is partially dominant (4–14% survival in heterozygous mosquitoes). Rapid fixation occurs because the initial frequency of matings among adults carrying Ile1,016 is expected to be small (0.14% in Fisher's model). There is also an extreme selection differential among mosquitoes because while the frequency of Ile1,016 homozygous and heterozygous mosquitoes are low (1.37×10^−7^ and 7.4×10^−4^ respectively in Fisher's model), most of the population is killed off by the insecticide. Thus, the frequency of Ile1,016 in the next generation becomes high because only homozygous mosquitoes and from 4–14% of the heterozygous mosquitoes survived.

Figure 5 also presents a third scenario wherein, following permethrin exposure, mosquitoes recover over the next few hours from the knockdown. This was measured by removing mosquitoes from the bioassay bottle to an insecticide free cage [24],[25]. We observed that all Ile1,016 homozygotes, 58% of heterozygotes and 15% of Val 1,1016 homozygotes recovered. Under this set of fitness conditions, Ile1,016 goes to fixation more slowly than when Ile1,016 is almost completely recessive.

## Discussion

The primary findings of this study are that: (1) frequencies of Ile1,016 in collections of the dengue virus vector *Ae. aegypti* have increased dramatically in the last decade in several states in México including Nuevo León to the north, Veracruz on the central Atlantic Coast and Chiapas, Quintana Roo, and Yucatán in the south; and (2) there was a strong association between the frequency of Ile1,016 homozygotes in a collection and knockdown rate in a bioassay. This complements earlier work [24],[25] which documented that, in bottle bioassays with 5 or 10 µg permethrin, Ile1,016 segregates as a recessive allele conferring complete knockdown resistance in homozygous mosquitoes, whereas there is 86–96% mortality in heterozygous mosquitoes and complete mortality in Val1,016 homozygous mosquitoes.

The analysis of predicted Ile1,016 frequencies using Fisher's model (Figure 5) was included only to illustrate that a simple model of selection predicts the rapid increases in Ile1,016 frequencies that we have observed. Fisher's model assumes a closed population of infinite size that is uniformly exposed to selection. In reality, we find extensive gene flow among all collections within 130 km of one another in northeastern México and within 180 km of one another in the Yucatán [27]. Susceptibility alleles are therefore probably continuously reintroduced into treated populations. Further, permethrin applications are not uniform in and among cities and towns in México. Thus through long distance transport of *Ae. aegypti* during human commerce, local mosquito migration, and variable levels of permethrin exposure in space and time, there is ample opportunity for recruitment of Val1,016 homozygotes into a population. Another major caveat to the model used in Figure 5 is that it assumed that Ile1,016 confers the same marginal fitness in the absence of permethrin. In fact, we have observed that it was easy to select Ile1,016 homozygous strains in the laboratory but very difficult to maintain them due primarily to egg and early larval instar mortality. It is also interesting that Ile1,016 frequency declined in the state of Quintana Roo between 2007 and 2008 and in Ciudad Hidalgo between 2006 and 2008.

These observations are by no means definitive evidence of reduced fitness of Ile1,016 in permethrin free environments; the same results could have occurred through genetic drift in the field or through the concentration of deleterious and lethal recessive genes in strains during selection for Ile1,016 homozygous strains in the laboratory. Nevertheless, several studies have documented negative fitness effects associated with single-point mutations in *para* that confer kdr to pyrethroids and DDT. For example, behavioral studies on peach–potato aphids (*Myzus persicae*) showed that a reduced response to alarm pheromone was associated with both gene amplification and a *para* target-site mutation [40]. In *Musca domestica*, flies with the identical *para* mutations showed no positional preference along a temperature gradient while susceptible genotypes exhibited a strong preference for warmer temperatures [41]. Studies of *para* in *Drosophila melanogaster* link point mutations to behavioral disturbances. For example, the temperature-sensitive paralysis exhibited by individuals carrying the point mutation *napts* (no action potential, temperature sensitive) resulted from a reduction in the expression of the *para* gene [42]. This is consistent with a model in which as temperature rises, an increasing fraction of the available sodium channels are required to maintain propagation of action potentials. Fewer channels cannot meet the demands of elevated temperature [43]. The mutation *tipE* (temperature-induced paralysis locus E) also disrupts *para* expression and confers temperature induced paralysis as a result of a decrease in sodium channel numbers [44],[45]. The *sbl* (smellblind) mutation, is also an allele of the *para* gene [46]. This associates sodium channel mutations with olfactory and chemotactic defects [46]–[48] and with changes in sexual behavior [48],[49].

A 1986 National Research Council report on strategies and tactics for pesticide resistance management [50], concluded that insecticide susceptibility should be viewed as a “natural resource” at risk of depletion if not managed properly. This lead to the concept of insecticide resistance management (IRM) [51]–[54]. One example of the value of implementing IRM schemes for management of mosquito vectors comes from a large-scale field demonstration project in southern Mexico that compared insecticide rotations (carbamates, organophosphates and pyrethroids) and insecticide mosaics (organophosphates and pyrethroids) with single use of insecticides (DDT or pyrethroids) for indoor residual spraying against anopheline malaria vectors [55]–[58]. This project demonstrated that use of insecticide rotations and mosaics, compared to single use of pyrethroids, can reduce resistance to pyrethroid insecticides in anophelines. If the same protocol could be implemented in *Ae. aegypti* then spatial refugia of susceptible *Ae. aegypti* or rotational schedules of different classes of adulticides could be established to slow or prevent fixation of Ile1,016. However we recognize that this strategy is ethically more applicable to agricultural pests than to disease vectors because people living in refugia may be at greater risk for acquiring DENV infections.

A large literature exists on the repellant properties of pyrethroids [59]–[64]. Their repellency led to the invention and deployment of pyrethroid-treated materials (curtains, screens and wall hangings) in the household. Female *Ae. aegypti* are endophagic and endophilic vector vectors and are almost exclusively anthropophilic [65]. Pyrethroid-treated materials may repel infected female *Ae. aegypti* from households and thus block DENV transmission in the household both by preventing inhabitants from becoming infected and from allowing infected inhabitants from transmitting DENV to *Ae. aegypti* in the home. Pyrethroid-treated materials used as curtains dramatically reduced *Ae. aegypti* populations and reduced DENV transmission in intervention versus control homes in Viet Nam [66]–[68], the Philippines [69], and Mexico and Venezuela [70]. A critical question is whether the Ile1,016 mutation reduces sensitivity to the repellant effects of pyrethroids. Consequently it is unknown as to whether kdr impacts indoor abundance of dengue virus-infected *Ae. aegypti* and dengue incidence. It is also unknown whether pyrethroid-treated materials will promote evolution of kdr in *Ae. aegypti*. It is likely that the current rise in Ile1,016 was driven by space spraying of pyrethroids to control adults in and around homes and non-target application of agricultural pyrethroids.

We have presented a retrospective study of the prevalence of the Ile1,016 mutation in natural populations of *Ae. aegypti*. It will now be important to begin prospective studies in México. Intensive studies of Ile1,016 at single sites may reveal the intensity of selection at these sites. Identification of cities or sites that are moving away from use of permethrin-based insecticides may enable us to explore negative fitness effects associated with the Ile1,016 mutation. These are not only academic exercises because as pesticides are applied and the target population becomes resistant, the susceptibility resource is depleted [71],[72]. A key assumption of IRM is that resistance alleles confer lower fitness in the absence of insecticides. Thus when a specific insecticide is discontinued, resistance will decline, and renew susceptibility. With sufficient time, during which alternative types of insecticides are used, the original insecticide can once again be applied. Resistance surveillance is an essential part of IRM schemes that provide data to inform program planning and pesticide selection, especially by detecting developing resistance at an early stage so that alternatives can be implemented.

The results presented here suggest that widespread spatial spraying of permethrin may be rapidly increasing the frequency of the Ile1,016 mutation in *Ae. aegypti* in México. This raises the question of whether permethrin-based insecticides should be replaced with other alternatives to maintain and restore susceptibility to pyrethroids. In addition to potentially improving vector control performance in the short term, this action also could protect the downstream potential for use of emerging vector control products impregnated with pyrethroids such as long-lasting textiles [15],[16],[73]. We do recognize the difficulties surrounding operational large-scale changes of insecticide use patterns but hope that our findings will help to inform the debate regarding the critical need to monitor and manage insecticide resistance in order to protect the limited options that are available to combat *Ae. aegypti* and reduce dengue.
